# Safety of Immune Checkpoint Inhibitors in Hepatitis B Virus-Positive Cancer Patients: A Multicenter Retrospective Cohort Study

**DOI:** 10.3390/curroncol33080469

**Published:** 2026-08-06

**Authors:** Meshail Baswaid, Alaa Shahbar, Afnan Noor, Danyah Ahmed Katlan, Abdulfattah Alhazmi, Mohammed Alnuhait, Aryaf Alsulami, Baker Saemaldaher, Hussam Magliah

**Affiliations:** 1 Pharmaceutical Care Department, International Medical Center Hospital, Jeddah 23214, Saudi Arabia; 2 Pharmaceutical Practices Department, College of Pharmacy, Umm Al-Qura University, Makkah 21955, Saudi Arabia; 3 Pharmaceutical Care Department, King Faisal Specialist Hospital & Research Center, Jeddah 22234, Saudi Arabia; 4 Pharmaceutical Care Department, King Fahad Armed Forces Hospital, Jeddah 23311, Saudi Arabia; 5 Department of Clinical Pharmacy, College of Pharmacy, Shaqra University, Al-Dawadmi 11961, Saudi Arabia; 6 College of Medicine, King Saud Bin Abdulaziz University for Health Sciences, Jeddah 21423, Saudi Arabia

**Keywords:** immune checkpoint inhibitors, hepatitis B virus, HBV reactivation, antiviral prophylaxis, cancer immunotherapy

## Abstract

Background: Immune checkpoint inhibitors (ICIs) are widely used in cancer treatment, but their safety in patients with pre-existing hepatitis B virus (HBV) infection remains uncertain. HBV reactivation is a recognized complication of immunosuppressive cancer therapy, yet evidence supporting routine antiviral prophylaxis during ICI treatment is limited. This study evaluated HBV reactivation and HBV-related outcomes among HBV-positive cancer patients receiving ICIs. Methods: This multicenter retrospective cohort study included adult patients treated with ICIs between January 2017 and December 2022. Patients were grouped according to receipt of antiviral prophylaxis. The primary outcome was HBV reactivation during ICI therapy. Secondary outcomes included hepatic flare, severity, and timing of reactivation, response to antiviral therapy, and factors associated with HBV-related outcomes. Results: A total of 160 patients were included; 68 received antiviral prophylaxis and 92 did not. HBV reactivation occurred in 3 patients (1.9%) and the odd ratio between antiviral prophylaxis and HBV reactivation was wide and imprecise (2.76; 95% CI 0.25–31.05). Hepatic flare occurred in 16 patients (10.0%) and was more frequent in the prophylaxis group than in the no-prophylaxis group (19.1% vs. 3.3%; *p* = 0.001). Conclusion: In this multicenter retrospective cohort study, the HBV reactivation rate was low and could not determine whether antiviral prophylaxis reduced the risk of reactivation. Hepatic flares were significantly more common among patients who received antiviral prophylaxis, likely reflecting a higher baseline risk of HBV-related complications in this group. A future randomized controlled trial is warranted to guide risk-adapted antiviral prophylaxis and monitoring.

## Simple Summary

Immune checkpoint inhibitors are evolutionary cancer treatment that enhance immune system to eradicate cancer cells. However, limited data are available regarding their safety in patients with hepatitis B infection, particularly the risk of hepatitis B viral reactivation. This retrospective cohort study included 160 cancer patients with chronic or past hepatitis B infection who received Immune checkpoint inhibitors. Patients were grouped according to receipt of antiviral prophylaxis. Among 160 patients, three patients developed hepatitis B viral reactivation. However, because of the small sample size and low event rate, the effect of antiviral prophylaxis on reducing reactivation risk could not be certainly determined. Alanine aminotransferase elevation, referred to as hepatic flare, occurred more frequently among patients who received antiviral prophylaxis. This outcome finding could be attributed to differences in baseline risk, close monitoring, and other confounding factors rather than harm from antiviral therapy. Future prospective studies are warranted to guide risk-adapted antiviral prophylaxis and monitoring in patients receiving immune checkpoint inhibitors, considering risk factors such as baseline hepatitis B viral DNA, hepatitis B surface antigen, type of malignancy, and concomitant immunosuppressant therapy.

## 1. Introduction

Immune checkpoint inhibitors (ICIs) are evolutionary cancer treatment option in addition to conventional chemotherapy, targeted therapy, and radiation therapy [1]. Progression-free survival and overall survival benefits of ICIs have been proven in randomized controlled trials, supporting their increasing use across various oncological indications [2]. The ICIs can provide durable clinical responses that may continue after treatment completion, which has expanded their use beyond metastatic disease to earlier cancer stages [3]. However, patients with chronic viral infections, including hepatitis B virus (HBV), were not adequately represented in many clinical trials. Therefore, the safety of ICIs in patients with pre-existing HBV infection remains uncertain, leaving healthcare providers with limited evidence to guide treatment decisions in this population [4].

ICIs enhance antitumor immunity by restoring T-cell activity through blockade of inhibitory immune checkpoints. However, this immune activation may also lead to immune-mediated adverse events, including hepatitis [1]. In patients with HBV infection, nucleos(t)ide analogs suppress the HBV replication and rarely achieve HBsAg seroconversion, and thus cancer patients remain at risk of HBV reactivation during systemic anticancer chemotherapy [5,6].

Available clinical evidence regarding HBV reactivation during ICI therapy remains limited. A study of hepatocellular carcinoma patients receiving nivolumab included 50 patients with HBV infection and reported similar 6-month overall survival between HBV-infected and uninfected patients, at 84% and 83%, respectively. However, the objective response rate was lower among patients with HBV infection compared with uninfected patients, at 14% versus 23%. Notably, none of the HBV patients had HBV reactivation nor anti-HBs seroconversion. In that study, patients with HBV were required to receive antiviral therapy and have an HBV viral load of less than 100 IU/mL before treatment initiation [7]. Nevertheless, the small sample size may have limited the study’s ability to detect meaningful differences between the two groups.

Another retrospective study included 114 patients with HBV infection who received ICIs for different solid tumors. Among them, 85 patients received antiviral prophylaxis, and 35 had detectable HBV DNA at baseline. HBV reactivation occurred more frequently in patients who did not receive antiviral prophylaxis compared with those who did, 5/29 versus 1/85 patients, respectively (odds ratio [OR] 17.50, 95% CI 1.95–157.07; *p* = 0.004). Moreover, none of the patients who developed HBV reactivation had a detectable HBV DNA at baseline. HBV-related hepatitis was also significantly more common in patients without antiviral prophylaxis, occurring in 4/29 patients compared with 1/85 patients receiving antiviral therapy (OR 13.44, 95% CI 1.44–152.79; *p* = 0.019) [8]. Despite the limited clinical evidence, antiviral prophylaxis may reduce HBV-related complications in patients receiving ICIs.

Based on this study and the reported signals of HBV reactivation in case reports, the American Society of Clinical Oncology (ASCO) recommends antiviral prophylaxis for HBsAg-positive patients receiving ICIs. In contrast, patients who are HBsAg-negative with past HBV infection should be closely monitored, unless they are receiving high-risk anticancer therapy [9]. Although ASCO has addressed HBV management in patients treated with ICIs, other guidelines have not provided specific recommendations for antiviral prophylaxis in this setting, underlining the ongoing ambiguity in clinical practice [8].

Most evidence on the safety of ICIs in HBV-positive cancer patients comes from East Asian cohorts and studies involving patients with hepatocellular carcinoma, where HBV prevalence, disease burden, and underlying liver disease may differ from those in other regions [10]. Due to the expanding use of ICIs in various malignancies, their safety in patients with HBV infection is tremendously important. Therefore, we aimed to demonstrate HBV reactivation rate and hepatic flare in patients with and without antiviral prophylaxis in this multi-center retrospective cohort study.

## 2. Methods

### 2.1. Study Design and Setting

This multicenter retrospective cohort study was conducted at three tertiary care hospitals in Jeddah, Saudi Arabia: King Fahad Armed Forces Hospital, King Abdulaziz Medical City, and King Faisal Specialist Hospital and Research Center. The study included patients who received immune checkpoint inhibitors (ICIs) between 1 January 2017, and 31 December 2022.

### 2.2. Study Population

Adult patients aged 18 years or older were eligible if they had received at least one dose of an ICI and had evidence of current or prior HBV infection, defined as positive hepatitis B surface antigen (HBsAg) and/or positive hepatitis B core antibody (anti-HBc). Patients were categorized according to whether they received antiviral prophylaxis during ICI therapy.

Patients were excluded if they were younger than 18 years, pregnant, had systemic autoimmune disease, were receiving chronic immunosuppressive therapy, were receiving prednisone-equivalent doses of ≥10 mg/day, or had received high-risk immunosuppressive therapies, including anti-CD20 monoclonal antibodies or anthracycline-based therapy.

### 2.3. Data Sources and Collection

Data were collected from electronic medical records included demographic characteristics, comorbidities, cancer type, ICI, antiviral prophylaxis status, liver function tests, HBV serologic markers, HBV DNA levels, and clinical outcomes.

Baseline variables included age, sex, body mass index, diabetes mellitus, chronic kidney disease, liver cirrhosis, alanine aminotransferase, aspartate aminotransferase, and total bilirubin. Treatment-related variables included the type of ICI received and the use of antiviral prophylaxis.

### 2.4. Definitions

In HBsAg-positive patients with detectable HBV DNA at baseline, HBV reactivation was defined as a ≥100-fold increase in HBV DNA from baseline. If HBV DNA was undetectable at baseline, HBV reactivation was defined as an increase in HBV DNA to ≥1000 IU/mL. If baseline HBV DNA testing was not performed, HBV reactivation was confirmed when HBV DNA became ≥10,000 IU/mL. In HBsAg-negative patients with positive hepatitis B core antibody, HBV reactivation was defined as HBsAg seroreversion or detection of HBV DNA. These definitions were based on the American Association for the Study of Liver Diseases 2018 guidance.

Hepatic flare was defined as an alanine aminotransferase elevation greater than two to five times the baseline value or the upper limit of normal. Immune-mediated hepatitis was assessed based on liver enzyme elevation according to immunotherapy-related toxicity grading criteria.

### 2.5. Outcomes

The primary outcome was the rate of HBV reactivation in patients receiving ICIs, with comparison between patients with and without antiviral prophylaxis. Secondary outcomes included hepatic flare, severity of HBV reactivation, time to HBV reactivation, response to antiviral therapy after reactivation, and the association between ICI type and HBV-related outcomes.

### 2.6. Statistical Analysis

Descriptive statistics were used to summarize baseline demographic and clinical characteristics. Continuous variables were reported as mean ± standard deviation or median with interquartile range, as appropriate. Categorical variables were reported as frequencies and percentages.

Baseline characteristics and outcomes were compared between the prophylaxis and no-prophylaxis groups using Pearson’s chi-square test, Fisher’s exact test, or the Wilcoxon rank-sum test, as appropriate. Statistical significance was defined as *p* < 0.05.

## 3. Results

### 3.1. Study Population

A total of 160 patients receiving ICIs were included in the analysis. Among them, 68 patients (42.5%) received antiviral prophylaxis, and 92 patients (57.5%) did not receive prophylaxis.

### 3.2. Baseline Characteristics

Baseline demographic and clinical characteristics stratified by antiviral prophylaxis status are summarized in Table 1. Patients in the prophylaxis and no-prophylaxis groups were comparable in terms of age, body mass index (BMI), and baseline liver biochemistry, including alanine aminotransferase (ALT), aspartate aminotransferase (AST), and total bilirubin. There were no statistically significant differences between groups with respect to gender, liver cirrhosis, or chronic kidney disease (CKD). However, diabetes mellitus was significantly more prevalent among patients receiving antiviral prophylaxis compared with those not receiving prophylaxis [χ^2^(1) = 7.65; *p* = 0.006], suggesting a higher burden of metabolic comorbidity in the prophylaxis group (Table 1). Among the 68 patients who received antiviral prophylaxis, 64 received entecavir, 3 received tenofovir disoproxil fumarate, and 1 received tenofovir alafenamide.

### 3.3. HBV Reactivation

Overall, HBV reactivation was rare, occurring in 3 of 160 patients (1.9%). Reactivation occurred in 2 patients (2.9%) receiving antiviral prophylaxis and 1 patient (1.1%) without prophylaxis (Table 2). The estimated odds ratio did not demonstrate a favorable effect of prophylaxis (OR = 2.76; 95%CI: 0.245–31.051), and confidence intervals were wide due to the small number of events. Because the very low number of events and the wide confidence interval, our study was underpowered to determine whether antiviral prophylaxis reduced the risk of HBV reactivation. Therefore, the odd ratio should be interpreted with caution and should not be considered evidence against the potential benefit of prophylaxis in higher-risk patients.

### 3.4. Hepatic Flare

Hepatic flare occurred in 16 patients (10.0%) in the overall cohort. Flare was significantly more common among patients receiving antiviral prophylaxis compared with patients not receiving prophylaxis. In the prophylaxis group, 13 of 68 patients (19.1%) developed hepatic flare, compared with 3 of 92 patients (3.3%) in the no-prophylaxis group (Table 3).

The unadjusted analysis showed increased odds of hepatic flare among patients receiving prophylaxis [OR of 7.01, 95% CI 1.91–25.72]. The odd ratio estimate cannot be certain in the presence of low hepatic flare events and wide confidence of interval. Also, this finding may reflect differences in baseline HBV risk, clinician selection for prophylaxis, or closer laboratory monitoring in patients perceived to be at higher risk.

### 3.5. Type of Immune Checkpoint Inhibitor

The type of ICI was significantly associated with HBV reactivation in unadjusted analysis (*p* = 0.008). However, interpretation is limited because the number of reactivation events was small and several ICI categories had low expected frequencies. No significant association was observed between ICI type and hepatic flare (*p* = 0.768).

## 4. Discussion

In this multicenter retrospective cohort study, the rate of HBV reactivation in patients receiving ICIs was uncommon, representing 1.9% of the cohort. This suggests a low risk of HBV reactivation in patients receiving ICIs, specifically in those who are not receiving a highly immunosuppressive therapy such as anti-CD20 monoclonal antibodies, anthracyclines, or chronic steroid equivalent to prednisone doses of ≥10 mg/day.

Our findings are consistent with previous studies showing low rates of HBV reactivation among patients receiving ICIs. Studies evaluated patients with unresectable hepatocellular carcinoma treated with ICIs and found that HBV reactivation was uncommon, particularly in those receiving antiviral therapy [7,11]. Similarly, Tapia Rico et al. reviewed the available evidence on ICIs in patients with chronic viral infections and reported that HBV reactivation during ICI therapy appears to be rare when appropriate monitoring and antiviral management are implemented [12].

HBV reactivation rates reported in retrospective studies may differ between patients who receive antiviral prophylaxis and those who do not because of variations in patient populations, cancer types, underlying liver disease burden, and monitoring practices across hospitals [12]. In our study, the low number of HBV reactivation events limited statistical power; therefore, we could not determine whether antiviral prophylaxis reduced the risk of HBV reactivation. Previous retrospective analyses have reported lower rates of HBV reactivation among patients who received antiviral prophylaxis [8,10,13].

In addition, hepatic flare was more frequent in the prophylaxis group than in the no-prophylaxis group, occurring in 13 patients (19.1%) versus 3 patients (3.3%), respectively, with patients receiving prophylaxis having higher odds of hepatic flare (OR 7.01, 95% CI 1.91–25.72; *p* = 0.001). Nevertheless, this finding should be interpreted with caution, as the small number of reactivation events limits statistical power and increases uncertainty around the effect estimates. Moreover, antiviral prophylaxis may have been preferentially prescribed to patients considered at higher baseline risk for HBV-related complications. Also, ALT elevation should not be interpreted solely as HBV-related hepatic flare, as several other factors may contribute to increased ALT levels, including immune-mediated hepatitis related to ICIs, liver metastases, disease progression, or other underlying hepatic conditions, especially in the absence of a quantitative HBV DNA test performed at the peak of ALT elevation.

Several potential confounding factors were not fully captured in this study. Baseline HBV DNA testing was available for 35 (21.8%) patients, and information on liver cirrhosis, liver metastases, and the duration of follow-up was incomplete. For this reason, the higher rate of ALT elevations in the prophylaxis group should not be viewed as evidence that antiviral prophylaxis caused harm. It may instead be related to differences in baseline clinical risk, more frequent monitoring, or other unmeasured factors. These potential confounding factors may have influenced the occurrence of HBV reactivation and hepatic flare. Future studies are needed to better clarify the clinical and biological mechanisms underlying these findings.

Development of detectable HBV DNA during immunosuppressive or chemotherapeutic therapy was evaluated in a retrospective study that included 71 patients with HBsAg-positive. The aim was to distinguish transient HBV DNA reappearance from true HBV reactivation. Among patients who were initially monitored without nucleos(t)ide analog therapy 18 of 25 patients achieved spontaneous HBV DNA clearance, while 7 patients transformed to true reactivation, defined as HBV DNA ≥3.3 log IU/mL. True reactivation was associated with hematologic malignancies and in those with an initial HBV DNA level ≥2.0 log IU/mL [14]. These findings indicate that baseline and serial HBV DNA monitoring are important factors to detect HBV related outcomes. In our cohort, HBV DNA testing was not available for all patients, which limited our ability to distinguish true HBV reactivation from transient HBV DNA detectability or other causes of hepatic flare. Future prospective studies are needed to distinguish resolved HBV infection from chronic HBsAg-positive infection and to incorporate serial HBV DNA monitoring, which may help better define true HBV reactivation during ICI therapy.

Apart from the well-recognized high-risk settings for HBV reactivation, such as hematologic malignancies and the use of potent immunosuppressive therapies including anti-CD20-depleting agents, data on the risk of HBV reactivation with ICIs remain limited, particularly among patients with solid tumors and HBsAg-negative status. In a retrospective study of 150 patients with solid tumors who received ICIs, no cases of HBV reactivation were reported. In that cohort, 38 patients (25.3%) had potential occult HBV infection based on anti-HBc positivity [15]. Monitoring of HBsAg, and liver function test, every three months, are proposed for HBsAg-negative patients with occult hepatitis B infection. Antiviral of nucleos(t)ide analog therapy is suggested for seroconversion of HBsAg or HBV DNA level > 1000 IU/mL [16]. This is consistent with our findings, as no HBV reactivation events were observed among HBsAg-negative cohort. However, baseline and periodic HBV DNA testing during ICI therapy were not consistently available in our patients. Regular HBV DNA monitoring may help identify patients at higher risk of HBV reactivation and may also improve the ability to distinguish HBV-related hepatic flare from other causes of ALT elevation.

Immune-mediated transaminitis is common with the use of ICI which is characterized by elevation of liver function tests. It should be differentiated from HBV reactivation which is associated with an increase in HBV viral load. The management of immune-mediated hepatitis usually involves consideration of corticosteroid therapy, whereas HBV reactivation is managed with nucleos(t)ide analogs. This distinction is clinically important because corticosteroids may worsen unrecognized HBV reactivation if started without appropriate antiviral therapy [17]. Therefore, ALT elevation should not be considered an immune-mediated reaction without assessing HBV DNA test at the peak of ALT, autoimmune tests, and other liver function tests.

HBV reactivation in our study occurred in patients who were HBsAg-positive and HBeAg-positive while no reactivation event was observed among HBsAg-negative patients (Table 4). This is consistent with ASCO guidance, which recommends antiviral prophylaxis for HBsAg-positive patients receiving ICIs while close monitoring may be sufficient in HBsAg-negative/anti-HBc-positive patients who are not receiving immunosuppressive therapy associated with a high risk of HBV reactivation.

Our study provides additional real-world data on the safety of ICIs in patients with HBV infection across different malignancies in the Saudi population. ICIs may be considered in this population after careful assessment of HBV reactivation risk and the need for antiviral prophylaxis. In addition, our findings demonstrated that HBV reactivation was successfully managed in patients who developed reactivation despite single-agent antiviral prophylaxis. Liver function tests subsequently normalized after escalation of antiviral therapy, including treatment with entecavir or tenofovir.

Our multicenter retrospective cohort study provides real-world evidence from Saudi Arabia across a diverse range of malignancies. These data offer additional clinical insight from an underrepresented geographic region, where the existing evidence is largely dominated by East Asian cohorts and studies focused on hepatocellular carcinoma. However, the study has several limitations, including the small sample size, low number of HBV reactivation events, potential misclassification of hepatitis flares, missing quantitative baseline HBV DNA values, inability to assess antiviral adherence, and incomplete laboratory data at the time of ALT elevation. In addition to that, median follow-up duration, duration of ICI exposure, number of ICI cycles, and timing of antiviral initiation were not available.

## 5. Conclusions

In this multicenter-retrospective cohort study, the HBV reactivation rate was uncommon and could not determine whether antiviral prophylaxis reduced the risk of reactivation. Hepatic flares were significantly more common among patients who received antiviral prophylaxis, likely reflecting a higher baseline risk of HBV-related complications in this group. These findings highlight the importance of individualized clinical assessment of HBV reactivation risk, appropriate consideration of antiviral prophylaxis, and close monitoring of liver enzymes and HBV DNA during ICI therapy. A future prospective trial is warranted to guide risk-adapted antiviral prophylaxis and monitoring.

## Figures and Tables

**Table 1:** Baseline Characteristics According to Antiviral Prophylaxis Status.

Characteristic	Overall N = 160	No Prophylaxis N = 92	Antiviral Prophylaxis N = 68	*p*-Value ^1^
Sex, n (%)				0.3
Male	115 (71.9)	63 (68.5)	52 (76.5)	
Female	45 (28.1)	29 (31.5)	16 (23.5)	
Age (years), mean ± SD	67 ± 12	69 ± 12	65 ± 12	0.12
BMI (kg/m^2^), mean ± SD	25.1 ± 5.7	25.2 ± 5.9	24.9 ± 5.5	0.6
Diabetes mellitus, n (%)	52 (32.5)	38 (41.3)	14 (20.6)	0.006
Liver cirrhosis, n (%)	9 (5.6)	7 (7.6)	2 (2.9)	0.3
Chronic kidney disease, n (%)	11 (6.9)	6 (6.5)	5 (7.4)	>0.9
HBsAg-positive, n (%)	43 (26.9)	16 (17.4)	27 (39.7)	0.002
Baseline HBV DNA available, n (%)	35 (21.9)	7 (7.6)	28 (41.2)	<0.001

^1^ Pearson’s chi-square test, Fisher’s exact test, or Wilcoxon rank-sum test, as appropriate.

**Table 2:** Association Between Antiviral Prophylaxis and HBV Reactivation.

Outcome	No Prophylaxis (N = 92)	Antiviral Prophylaxis (N = 68)	Odds Ratio (95% CI)	*p*-Value *
HBV reactivation, n (%)	1 (1.1%)	2 (2.9%)	2.76 (0.25–31.05)	0.575

* Fisher’s exact test.

**Table 3:** Association between antiviral prophylaxis and hepatic flare.

Outcome	No Prophylaxis (N = 92)	Prophylaxis (N = 68)	Odds Ratio (95% CI)	*p*-Value
Hepatic flare, n (%)	3 (3.3%)	13 (19.1%)	7.01 (1.91–25.72)	0.001

**Table 4:** Patient characteristics, baseline HBV status, and outcomes following HBV reactivation during ICI therapy.

	Patient Characteristics				Baseline			Outcomes				
Patient	Age	Sex	ICI	Cancer Type	HBV DNA (IU/mL)	HBeAg	Antiviral Prophylaxis	Onset (Days)	VHI Grading	Peak ALT (U/L)	Outcome	Management
1	76	Male	Pembrolizumab	Lymphoma	N/A	Positive	TDF	141	V1, H2, I3	200	Normalized LFTs after 4 weeks	Entecavir + tenofovir
2	64	Male	Durvalumab	Lung cancer	156	Positive	Entecavir	90	V1, H2, I2	130	Normalized LFTs after 2 weeks	Entecavir + tenofovir
3	73	Male	Pembrolizumab	Lymphoma	N/A	Positive	Not on antiviral	390	V1, H2, I2	1119	Normalized LFTs after 4 weeks	Entecavir

ALT, alanine aminotransferase; DNA, deoxyribonucleic acid; HBeAg, hepatitis B e antigen; HBV, hepatitis B virus; ICI, immune checkpoint inhibitor; LFTs, liver function tests; N/A, not available; TDF, tenofovir disoproxil fumarate; ULN, upper limit of normal; VHI, viral hepatitis infection. V1: HBV DNA increase by >1 log IU/mL or change from undetectable to detectable. H2: ALT >10× ULN and/or bilirubin >2× ULN or INR >1.3. I2: Interruption of immunosuppressive therapy with re-initiation of the same agent after hepatitis flare resolution. I3: Interruption of immunosuppressive regimen with re-initiation of second-line or suboptimal therapy.

## Data Availability

The authors confirm that the data supporting the findings of this study are available within the article.
